# To Invest or Not to Invest, That Is the Question: Analysis of Firm Behavior under Anticipated Shocks

**DOI:** 10.1371/journal.pone.0158782

**Published:** 2016-08-10

**Authors:** Dejan Kovac, Vuk Vukovic, Nikola Kleut, Boris Podobnik

**Affiliations:** 1 CERGE-EI, A joint workplace of the Center for Economic Research and Graduate Education, Charles University, Prague, and the Economics Institute of the Academy of Sciences of the Czech Republic, Prague, Czech Republic; 2 Luxembourg School of Business, Luxembourg, Grand-Duchy of Luxembourg; 3 Department of Economics, Zagreb School of Economics and Management, Zagreb, Croatia; 4 Adriatic Economic Association, Zagreb, Croatia; 5 Zenlab d.o.o., Zagreb, Croatia; 6 Faculty of Economics, University of Ljubljana, Ljubljana, Slovenia; 7 Faculty of Civil Engineering, University of Rijeka, Rijeka, Croatia; East China University of Science and Technology, CHINA

## Abstract

When companies are faced with an upcoming and expected economic shock some of them tend to react better than others. They adapt by initiating investments thus successfully weathering the storm, while others, even though they possess the same information set, fail to adopt the same business strategy and eventually succumb to the crisis. We use a unique setting of the recent financial crisis in Croatia as an exogenous shock that hit the country with a time lag, allowing the domestic firms to adapt. We perform a survival analysis on the entire population of 144,000 firms in Croatia during the period from 2003 to 2015, and test whether investment prior to the anticipated shock makes firms more likely to survive the recession. We find that small and micro firms, which decided to invest, had between 60 and 70% higher survival rates than similar firms that chose not to invest. This claim is supported by both non-parametric and parametric tests in the survival analysis. From a normative perspective this finding could be important in mitigating the negative effects on aggregate demand during strong recessionary periods.

## Introduction

The pinnacle of the Schumpeterian creative destruction hypothesis is that successful adaptation to market fluctuations makes the crucial difference between firms that fail and firms that survive. Within-firm innovation and the capacity to adapt to the persistently ongoing process of technological change have widely been established as essential reasons of what makes a company robust and enduring. Some companies simply react better to technology and productivity shocks. In this paper we aim to test the response mechanism of companies when facing an upcoming and expected shock.

We focus on firm investment in long-term assets as one potential strategy a firm can use as a response to a shock. Investment in long-term assets is not only a sign of a firm’s favourable credit rating and access to finance, but more importantly, it sends a strategic signal that the firm is expecting to endure the upcoming shock. Essentially, we look at two managers from two identical firms where one invests and the other does not. Managers that do invest during the crisis obviously expect higher profits from investing than from non-investing. We are interested to see how this strategy to increase investment in long-term assets prior to the expected shock will affect a firm’s probability of survival.

**Fig 1:**
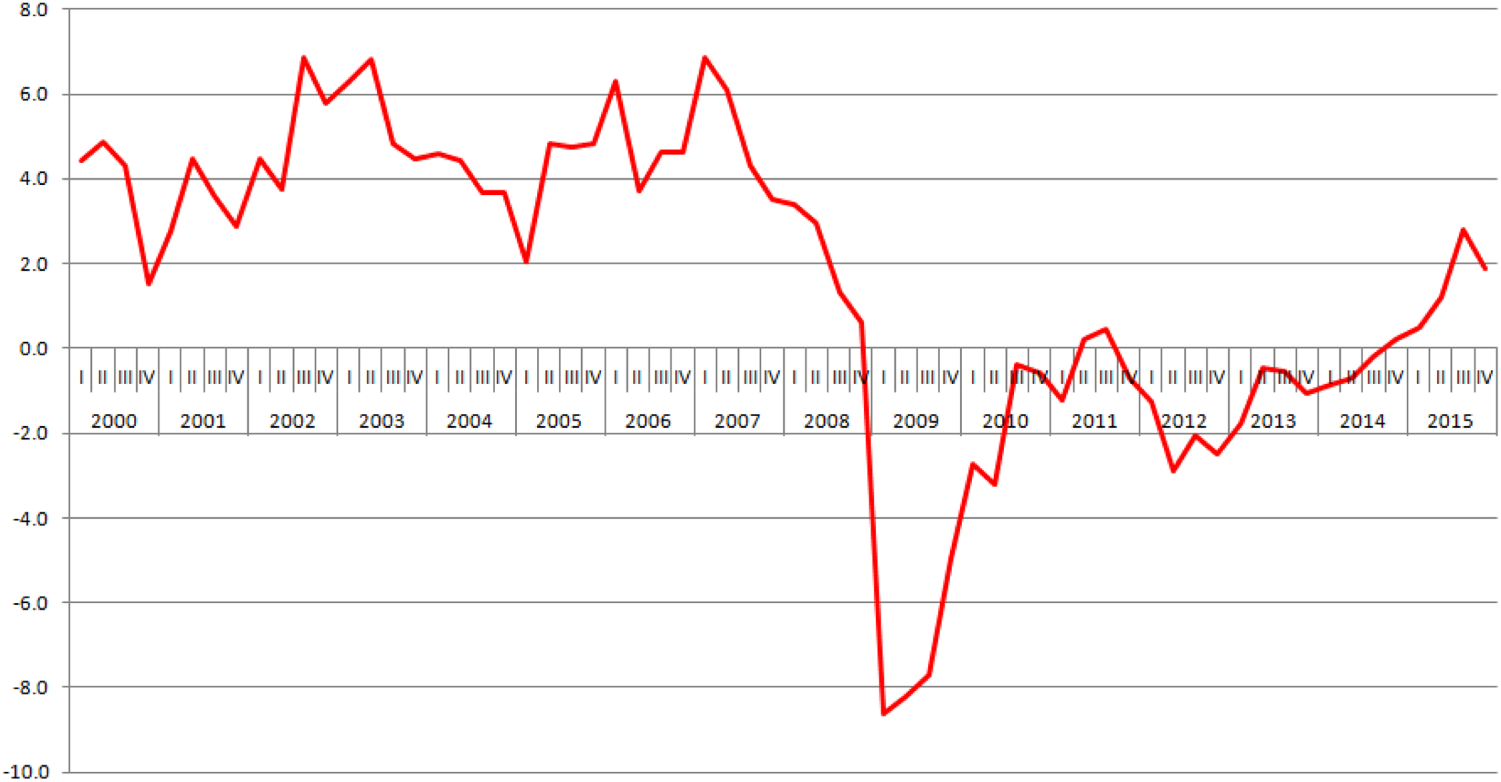
Croatian quarterly GDP growth rate, 2000-2015. Source of data: Croatian Bureau of Statistics (DZS, 2014).

We use the recent 2007-2009 financial crisis as a perfect exogenous shock that hit Europe and subsequently Croatia with a certain time lag. Following the burst of the housing market bubble in the United States in mid-2007, which quickly transcended into the finance industry with the collapse of Bear Stearns in March 2008, it can be inferred that the rest of the world, Europe in particular, was expecting the shock to spilover rather quickly onto its domestic markets. According to the news reports from the beginning of 2008, it was becoming clear the US has entered the recession as foreclosures in America spiked, while home sales started declining [1, 2]. In Croatia the first big decline in GDP came in the first quarter of 2009 (an 8.6% decrease), even though the stagnation could have already been felt in the final two quarters of 2008 [3]. We assume that the time lag since the burst of the US housing bubble and the consequential panic in the US banking industry in the second half of 2008 to the first real shock of the crisis in Croatia in the first half of 2009, was more than enough for domestic firms to properly anticipate and react to it by forming an optimal response strategy. Fig 1 shows the longevity of the crisis shock in Croatia, where the recession has been virtually interrupted for six years.

The economic intuition behind our claim is to compare anticipated with unanticipated shocks. Do anticipated over unanticipated shocks influence economic agents to re-optimize their decisions? There is a vast literature exploring the behavior of economic agents under anticipated and unanticipated shocks to the economy [4–6]. According to economic theory, agents base their decision rules upon their current information set about variables relevant for the decision problem. In Croatia we see that some companies reacted quite well to the signal of an upcoming crisis and stocked up in anticipation of the shock and the subsequent lending squeeze, while others were left hopelessly stranded and without access to finance making it quite difficult to survive the long-lasting recession. Information asymmetry about the upcoming shock was hardly an issue differentiating these two groups as all firms had to have seen it coming by the second half of 2008. It is easy to attribute this argument to hindsight bias, however many Croatian firms in 2008 were actually expecting a slump. In a survey of business expectations published in the final quarter of 2008, out of 570 surveyed firms, over 90% of them said that they expect a crisis and a difficult year ahead [7].

We observe the differences in how firms reacted to this exogenous shock. We use the data for over 140,000 firms from 2003 to 2015, representing the entire population of Croatian firms, to examine the impact of the response strategy the firms used. We do this by comparing the business strategies of investing vs. non-investing firms in 2008, to see what made the difference between firms that managed to survive the crisis and those that suffered in comparison. We compare identical firms in 2007 and look at their response strategies in 2008 (the ‘adjustment period’ year). There is an obvious self-selection problem in this case as the probability of survival will most likely be correlated with the decision to invest. We solve this endogeneity problem by applying a *matching strategy* where we match investing and non-investing firms into random samples based on several firm characteristics: size of assets, total capital, total revenues, and credit exposure. We divide the firms so that their mean values are almost the same thus ensuring that we only observe similar firms. Additionally, our rich dataset (S1 Dataset) allows us to control for many important variables on firm level such as type of industry, type of investment, debt ratios, origin etc.

We hypothesize that firms which were able to successfully react to the anticipated shock increased their relative investments in long-term assets (relative to size of assets) in 2008, while firms that eventually went bankrupt (or have increased their probability of default) did not increase their investments and hence did not respond properly to the anticipated shock. Out results confirm this finding for micro and small firms: investing firms were clearly more successful in surviving the crisis. Our matching strategy in addition to a series of controls for other factors that might affect firm survival, ensure that our effects on firm survival were not driven by any factor other than investments in long-terms assets.

A normative implication would be to suggest to firms, particularly small and micro, not to delay or postpone investments while anticipating a crisis shock. On the contrary, investing prior to the shock sends a signal of strenght, proper planning, and endurance. Having more firms apply this kind of strategy can perhaps even help countries in mitigating the shock of the crisis. If firms are well-prepared and engaged in long-term projects once the crisis starts, they have less incentives to lay off workers, which could reduce the depression in aggregate demand. This will, on the supply side, also be better for firms as they won’t see their sales going down as much.

In addition to these main findings we also present a full duration analysis of the population of Croatian firms during the entire recessionary period. This is a pioneering analysis of this kind in Croatia. The paper is organized as follows: after the literature and data sections, we first present the results of the survival analysis for all Croatian firms during the crisis (applying both the non-parametric and parametric models), and then we test our investing vs. non-investing hypothesis with respect to firm size.

## Literature Review

In the most simplified form survival analysis observes the time duration of a subject until the occurrence of a particular event of interest. It originates from medical research where the event of interest is death, and engineering where the event represents a failure of a mechanical system (in engineering this is called reliability analysis). In economics the term used is duration analysis and its application is generally done in the theories of industrial organization. The event is firm bankruptcy, while the time to event is the period for which the firm is operational. The most interesting part behind duration analysis of firms is to try and figure out which characteristics make firms more or less robust to exogenous shocks, and what in general makes a company successful and enduring. In other words, duration analysis in economics reveals the ‘black box’ of the creative destruction process.

Accordingly, duration analysis of firm survival has mounted a rich body of empirical evidence [8–18]; insomuch that some of the main findings in the literature were given an attribute of stylized facts of firm survival.

The first stylized fact, proven by virtually every study of firm survival analysis, is that size tends to have a strong, positive impact on survival [8, 13, 16, 17, 19]. Even for start-up companies, larger start-ups (in terms of employment size) are more likely to expand, and thus survive, than smaller ones [20]. By the same rationale, as it grows larger and larger thus increasing its chances of survival, it makes sense for age to be positively correlated to firm survival [13]. Younger companies and start-ups usually have higher hazard rates and are more prone to initial failure, even though the relationship between firm survival and age is non-monotonous, and most likely concave (survival rate increases with age at first only to decline later).

Survival also depends on the industry sector the firm is a part of [8], where firms in expanding and up-and-coming sectors (such as the IT industry) have greater potential and thus greater survival rates. Export activity is yet another factor that affects firm survival. Facing international-level competition forces export companies to adapt faster and increase both their productivity and efficiency. Naturally such firms have a greater probability of survival [21–23].

Finally, firms that are more innovative—in terms of both R&D investment and the ability to adapt to technological change—experience higher survival rates than firms that fail to adapt to new technologies [24–27]. Firms that innovate are not only more successful in adapting to new market conditions, but are also more able to maintain the competitive advantage on the market. However the relationship between innovation and survival is also non-monotonous. Innovation is characterized by uncertainty. It depends on a number of categories, such as the company’s initial level of technological endowment, whether or not the company is operating in a highly innovative industry, and the type of innovation—where innovation flows (such as investments in patents) can actually harm a company’s survival chances [27].

A series of other factors have also been found to affect firm survival, such as organizational structure [9], market growth [28], pre-entry experience of entrepreneurs [29], the rate of increase of employment, size of investment in the industry, size of industry, and the rate of new firms entering the industry [27], learning-by-doing capabilities [17], initial endowments and prior experience [30], firm-level heterogeneity, financial leverage, labor productivity and the industry capital-labor ratio [31].

In addition to firm-specific characteristics, firm survival has also been found to depend on the stage of the industry life cycle and some industry-specific factors such as the level of competition, predictability of demand, or the rate of industry growth and technological change [17]. For example, in the final stages of the life cycle the firm size implication is irrelevant, as is for products with low levels of technological intensity [13, 27]. The success of new market entrants also depends on the specific phase of the life cycle [30, 32, 33]. The life-cycle role is important to consider when discussing firm survival, since the process of firm survival and market entry and exit is a dynamic category interlinking with both technology and market demand, which are time-variant. This is why the stylized facts, even though they have been consistently proven to hold, should always be considered within the scope of technological progress and possible exogenous shocks that affect industries and thereby can prove to be even more important in determining why a firm failed.

## Data and Methods

We use the several data sources to form our main duration analysis variables. We use the Croatian Statistical Business Register to get the statistical unit identification number (SIN) for each of our subjects along with all the registration information of every single company. This data served as our main duration indicators, collected for the entire population of 144,074 Croatian companies for the time span of twelve years (2003-2015). We have excluded non-government organizations’ registration information, as well as the budgetary users’ registration information and have only focused on for-profit entities, both private and public.

The registration data made it possible for us to formulate several important variables. First of all the dates of registration and the dates of cancelation, with which we could determine the total duration in days, months and years for each of our subjects. This represented our time variable in the duration analysis. From these two indicators we could also form our main event variable—the failure indicator. If a company went bankrupt in any point of time during our observed period it was given an indicator value of 1 (occurrence of event). If not, it was given a value of 0 (it survived).

Using the same indicators we also formulated the variable “socialist origin”, which determines whether or not the company was founded before 1992, the year in which the privatization process started in Croatia, and when the legal entities accepted new market regulations. In our sample around 14,000 companies were founded before 1992, and were operational and building their markets during socialism.

Furthermore, from the same registry we formulated the official industry classification codes, whether or not the company was a start-up, their subject size (micro, small, medium and large), their legal form (companies, farms, crafts, or freelancers), in which of the 21 Croatian counties they are based, in which of the 556 Croatian municipalities they are based, the fraction of domestic equity, the fraction of foreign equity for each firm, and the ownership type of each company (public, private, mixed, former public, etc.).

For each of these companies we collected the financial statements from the Croatian Financial agency (FINA), the official public database on business entities. It should be noted that the sample varies from one year to the next, as not all companies reported their financial statements to the authorities every year. The companies in the sample are those that had their financial statements submitted for at least a single year. In general for every individual year we have between 80,000 and 90,000 companies reporting their financial statements. This number is different from the original 144,000 due to two reasons. The first obvious reason is the varying number of entry and exits each year, while the second reason is due to a nonexistent legal constraint at the time, when not all firms submitted their financial statements for each year. However there was no systematic pattern among the non-reporters because for the majority of observations we were able to fill in the missing data using the end of the day balance of the previous year.

From the financial statements data we pulled out a variety of indicators that we used in our matching procedure of merging the two databases (S1 Dataset). We used long term assets (tangible and intangible, financial), current assets (inventories and receivables), total profit or loss, personnel costs, total capital and reserves, total loans, subsidies and net investment in fixed assets. We also calculated the changes for each of these categories from 2007 to 2008, as these are the two pre-crisis years that we observe.

Finally, we define a recession using the standard NBER definition where “a recession is a significant decline in economic activity that spreads across the economy and can last from a few months to more than a year” [34]. To identify a recession in Croatia we take the first three quarters of a consecutive decline of GDP. This coincided with the first three quarters of 2009 (Fig 1), which justifies our usage of 2007 and 2008 as pre-crisis years.

In the standard survival analysis the most important parameters are the failure event (in our case the bankruptcy of an observed firm), and the total timing until the occurrence of the event (the days for which the firm was able to continue with its business operations). Using our population we estimate the survival functions and hazard rates for our specified variables (non-parametric methods), after which we apply the widely used Cox proportional hazard model [35] (parametric method) to estimate the survival rates across some of the main variables we use.

We start by defining *T* as a non-negative continuous random variable representing the timing until the occurrence of an event (i.e. firm bankruptcy). It has a probability density function (p.d.f.) *f*(*t*) and a cumulative distribution function (c.d.f.) *F*(*t*) = *Pr*(*T* < *t*), defining the probability that the event has occurred by some duration *t*. From this we define the Kaplan-Meier [36] survival function in the following way:

$$\begin{array}{c} S\left(t\right)=Pr\left(T\geq t\right)=1-F\left(t\right)=\int_{t}^{\infty }f\left(x\right)dx \end{array}$$ (1)

Where *S*(*t*) is the probability that the event (firm bankruptcy) has not occurred before duration *t*.

In addition to the survival function we also use the hazard function which represents the rate of occurrence of the event.

$$\begin{array}{c} \lambda \left(t\right)=\lim_{dt\to 0}\frac{Pr(t\leq T<t+dt|T\geq t)}{dt} \end{array}$$ (2)

The numerator represents the conditional probability that the event will occur in the interval [*t*;*t* + *dt*) given that it has not occurred before, and the denominator represents the width of the interval. Dividing one by the other we obtain a rate of event occurrence per unit of time. Taking the limit as the width of the interval goes down to zero, we obtain an instantaneous rate of occurrence. Basically, the survival and the hazard functions provide alternative but equivalent characterisations of the distribution of *T*. We use and report each of these simultaneously in our analysis. Both of these functions represent non-parametric models of estimation.

The Cox [35] proportional hazard model on the other hand is a parametric estimation model. It focuses directly on the hazard function, where the hazard at time *t* for an individual firm with a set of covariates *x*_*i*_ is assumed to be:

$$\begin{array}{c} \lambda_{i}\left(t|x_{i}\right)=\lambda_{0}\left(t\right)exp\left\{x_{i}^{\prime }\beta \right\} \end{array}$$ (3)

In this model *λ*_0_(*t*) is a baseline hazard function that describes the risk for firms with *x*_*i*_ = 0, while $exp\{x_{i}^{\prime }\beta \}$ is the relative risk, a proportionate increase or reduction in risk, associated with the set of characteristics *x*_*i*_. The set of covariates we use is listed within each Cox model table we use. They represent different firm-specific characteristics for which we test firm survival.

Finally, we can integrate both sides from 0 to *t* to obtain the cumulative hazards:

$$\begin{array}{c} \Delta_{i}\left(t|x_{i}\right)=\Delta_{0}\left(t\right)exp\left\{x_{i}^{\prime }\beta \right\} \end{array}$$ (4)

which are also proportional. Changing signs and exponentiation we obtain the survivor functions:

$$\begin{array}{c} S_{i}\left(t|x_{i}\right)=S_{0}{\left(t\right)}^{exp\{x_{i}^{\prime }\beta \}} \end{array}$$ (5)

where the second part is the baseline survival function. Thus, the effect of the covariate values *x*_*i*_ on the survivor function is to raise it to a power given by the relative risk $exp\{x_{i}^{\prime }\beta \}$.

## Results

### Duration analysis for entire sample

**Fig 2:**
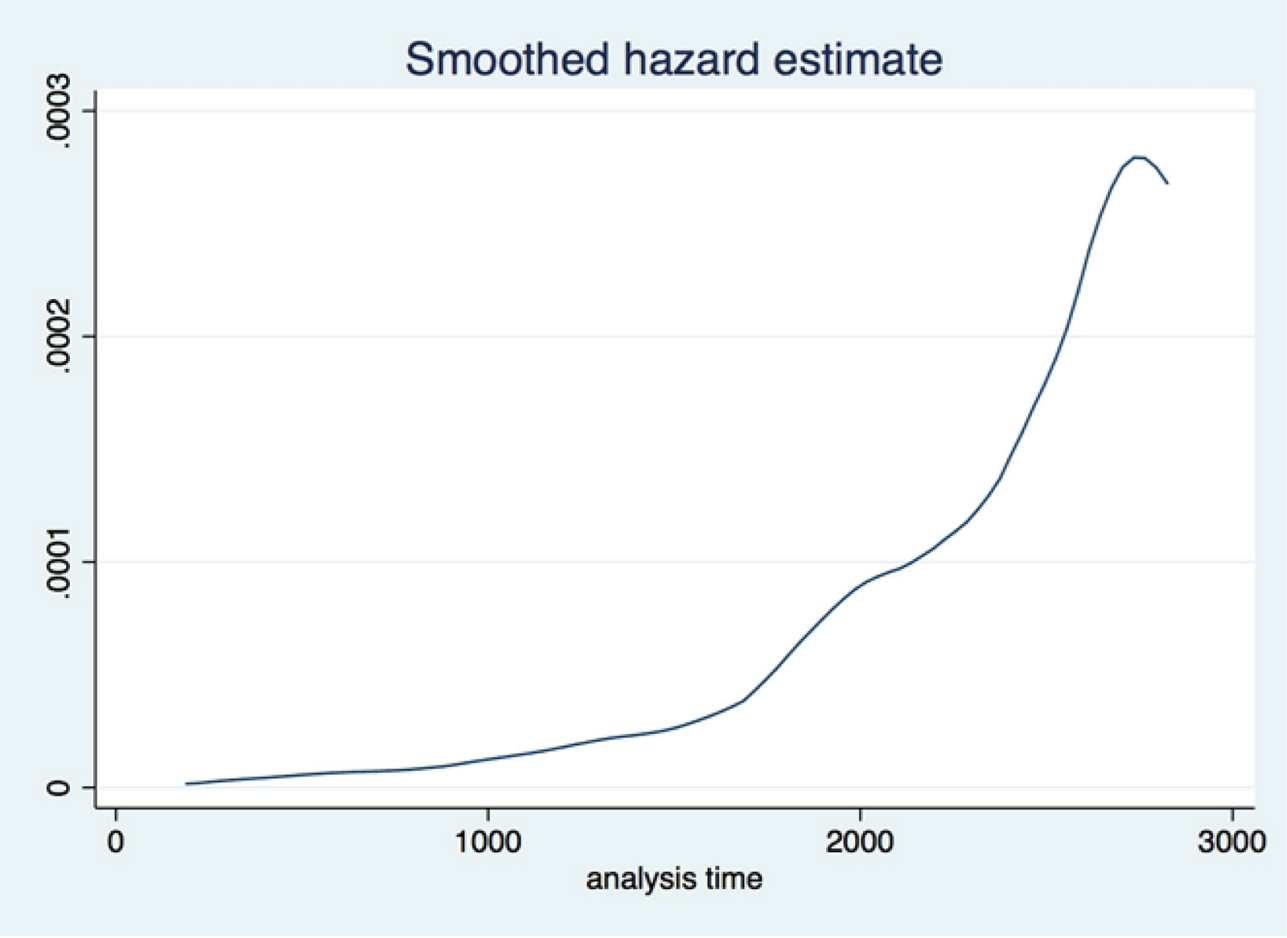
Hazard rate for all Croatian firms from 1.1.2007. to 31.3.2015.

**Fig 3:**
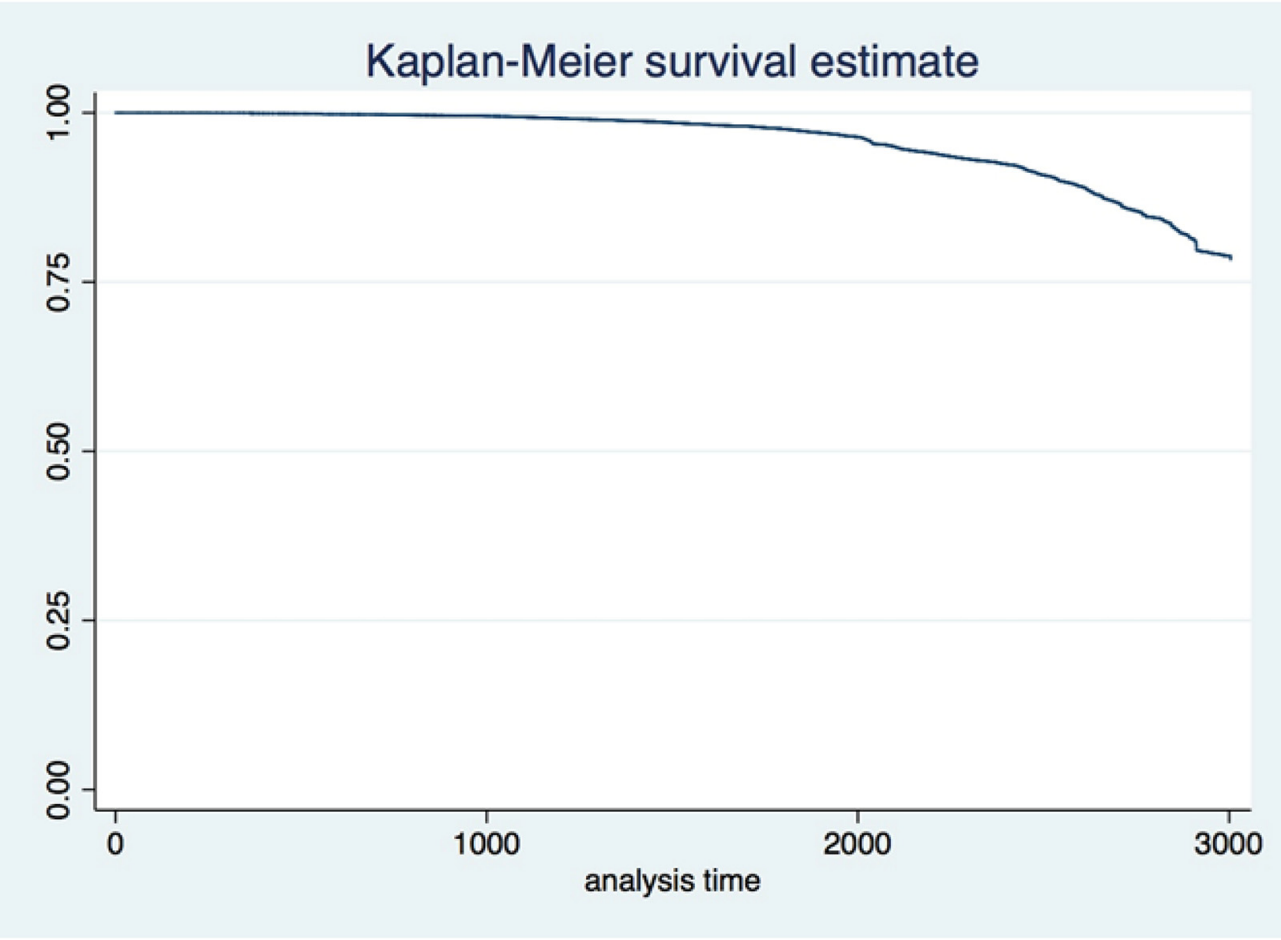
Kaplan-Meier survival estimate for Croatian firms from 1.1.2007. to 31.03.2015.

The initial analysis is focused on the economy as a whole, where we analyze 144,044 firms over an eight year period for which we have the available data. We employ two pre-crisis years (2007 and 2008) to calculate the survival rates before the crisis and compare it with the rates during the crisis. Fig 2 shows how the hazard rate within the economy is increasing as the economy enters the recession. Fig 3, which represents the Kaplan-Meier survival estimates, shows that from January 2007 to March 2015 the Croatian economy lost close to 25% of its firms. Both show an expected trend. However the hazard rate starts increasing around 2000 days. Our analysis looks at the data from January 2007, meaning that the crisis started somewhere around the 1000 days mark (first quarter of 2009), while firms did not start experiencing bankruptcies until 2012. This too makes sense since the initial reactions of mangers to the crisis shock is to lay off workers as business orders halt. It takes a while before the firms decide to close shop.

To check the validity of our duration analysis we employed an additional test for our independent variables. On a simple robust analysis we tested whether these variables are time-invariant. We calculated the firms’ average transition probability between industry sectors on a time span from 2007 to 2013 and found that they are roughly below 5 percent on average (S1 and S2 Tables), meaning that only a very small number of firms shifted between sectors. The number would be even smaller if we would test the transition probabilities on a year to year basis. Additionally we ran the same tests for county, size of firm, ownership type and legal form and we got even lower probabilities.

### Duration analysis by specific firm characteristics

**Fig 4:**
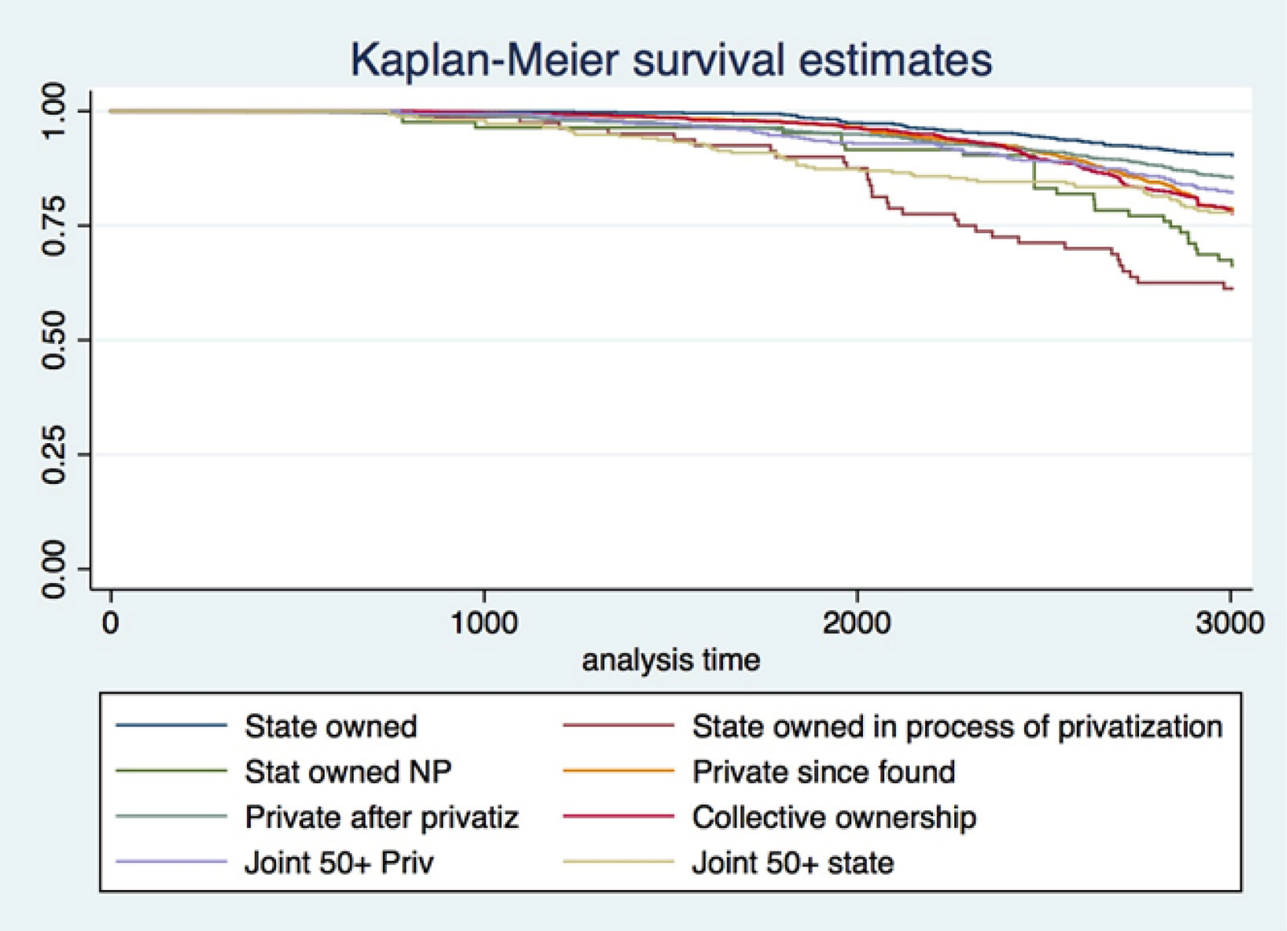
Kaplan-Meier survival estimate by type of ownership.

In order to be consistent with our methodology we were constrained only on analyzing firm characteristics that are time invariant. The characteristics that we used are ownership structure, origin of ownership, and firm size. We can see the structure of ownership across firms in Table 1 where, not surprisingly, the vast majority of firms (97.1%) are private since founding. The Kaplan-Meier survival estimate (Fig 4) shows that state owned firms have the highest survival rates, which is in line with the general predictions of economic theory.

**Table 1:** Distribution of firms by type of ownership.

Ownership type	Frequency	Percent	Cumulative
State ownership	850	0.59	0.59
State company in transformation	80	0.06	0.65
State company where transformation has not started	83	0.06	0.70
Private since founding	139,891	97.10	97.80
Private after transformation	1270	0.88	98.68
Cooperative ownership	1155	0.80	99.48
Mixed ownership with over 50% private capital	492	0.34	99.82
Mixed ownership with over 50% state capital	253	0.18	100
Total	144,074	100	—

State owned firms basically have no financial constraints and in times of recession their liquidity and solvency risks are on average lower than for private sector firms. Following the state owned firms, the second highest survival rate is attributed to firms that got privatized during the privatization process, which goes in line with the theory of mass privatization [37] where the most efficient firms are privatized first. On the other hand the worst performing companies were those that were state owned but still in the process of privatization. The enduring process of devaluing such firms has paid its toll during the current crisis. Finally, when we observe firms with joint public and private ownership we notice that firms with more than 50% state-owned shares have a lower survival rate than firms with a majority private ownership structure. This can be due to several reasons, where the main one could be a more efficient allocation of resources of firms under private sector control.

**Fig 5:**
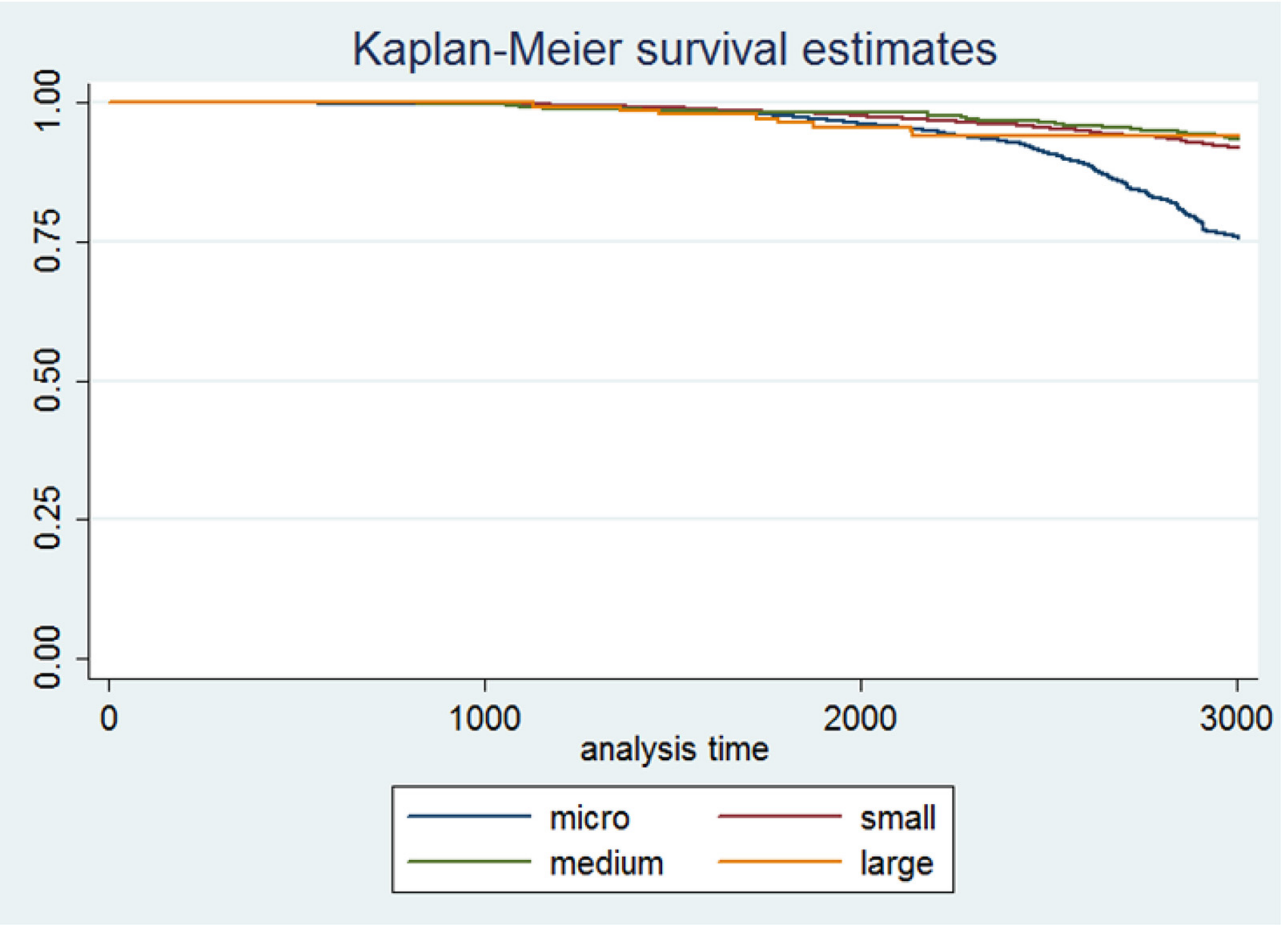
Kaplan-Meier survival estimate by firm size.

The final criteria is firm size. According to the classification in Table 2 we divided firms into micro, small, medium and large. The majority of the population are micro firms (Table 3), but as expected they have the smallest size of assets and revenues. The Kaplan-Meier survival estimates go in favor of big firms, where it is obvious that the larger the firm, the higher its probability of survival (Fig 5). In recessionary times this is usually correlated with having less financial constraints on further investments, a pattern which is observed in the data.

**Table 2:** Distribution of firms by type of ownership.

Size	Asset Criterion	Sales Criterion	Employee Criterion
Micro	<2,000	<2,000	<10
Small	<10,000	<10,000	10—49
Medium	<43,000	<50,000	49—250
Large	>43,000	>50,000	250>

The numbers for assets and sales are all in thousands of euros.

**Table 3:** Distribution of firms by type of ownership.

Size	Frequency	Percent	Cumulative
Micro	138,475	96.11	96.11
Small	4335	3.01	99.12
Medium	1009	0.70	99.82
Large	255	0.18	100
Total	144,074	100	—

### Cox proportional hazard model

#### All firms

Table 4 presents the Cox proportional hazard model estimates for the entire population. Instead of the usual hazard ratio it reports the calculated coefficients. It supports the results presented by the non-parametric methods regarding firm size, ownership type, and legal form; however we included a few additional variables to get a more complete picture of what drives firm survival in Croatia (or at least what affected firm survival during the crisis).

**Table 4:** Cox proportional hazard model—Full sample survival rates.

Variable	Parameter	Standard Error	P-value
Socialist origin	-0.101***	0.021	0.000
Start-up	-0.547***	0.013	0.000
Foreign	0.157***	0.021	0.000
**Legal form**			
Farms	-1.118***	0.193	0.000
Freelance	-1.175***	0.087	0.000
Craftsmen	0.195***	0.024	0.000
**Firm size**			
Small	-1.086***	0.051	0.000
Medium	-1.365***	0.121	0.000
Large	-1.487***	0.259	0.000
**Ownership type**			
State company in transformation	1.389***	0.211	0.000
State company where transformation has not started	1.232***	0.218	0.000
Private since founding	0.637***	0.111	0.000
Private after transformation	0.445***	0.132	0.001
Cooperative ownership	0.642***	0.126	0.000
Mixed ownership with over 50% private capital	0.679***	0.153	0.000
Mixed ownership with over 50% state capital	0.826***	0.173	0.000
**County**			
2	0.117**	0.049	0.018
3	0.335***	0.046	0.000
4	0.242***	0.045	0.000
5	0.119***	0.041	0.003
6	0.136***	0.052	0.008
7	0.226***	0.049	0.000
8	0.228***	0.029	0.000
9	0.093	0.075	0.214
10	0.23***	0.062	0.000
11	0.333***	0.066	0.000
12	0.266***	0.047	0.000
13	-0.033	0.042	0.416
14	0.413***	0.034	0.000
15	0.129***	0.046	0.005
16	0.441***	0.045	0.000
17	-0.111***	0.031	0.000
18	0.036	0.031	0.240
19	-0.077*	0.041	0.056
20	0.228***	0.041	0.000
21	0.046*	0.025	0.071
Observations	144,074		
LR test (prob>Chi2)	3775.87 (0)		

The table reports coefficients, not the hazard rates. A negative sign of the coefficient implies a lower hazard rate, i.e. higher probability of survival. The county variables represent each of the 21 Croatian counties, all listed in the standard legal order (where the first is Zagrebacka County (omitted), the second Krapinsko-Zagorska county, while the last one is the City of Zagreb). *** denotes significance at 1%, ** at 5%, and * at 10%

The first variable is socialist origin, which according to the estimates suggests that firms which were founded before the privatisation process in 1992 have a 10% higher probability of survival than firms founded later (note that the negative sign in the Cox regression implies a lower hazard rate, i.e. a greater probability of survival). This makes sense as most of these firms which were operational during socialism (and even before, founded in the beginning of the 20th century), have already built their markets and gained both customers and reputation earlier. It makes perfect sense for these companies to be more robust to crisis shocks. They have simply had, on average, more experience. The second variable is a dummy representing whether or not the firm was a start-up during the observed period. Contrary to the majority of the literature, we find that start-up companies have had a higher probability of survival, in this case 54.7%. This is most likely related to size: larger start-up firms have a greater probability of survival than smaller ones [20]. Once we separate firms according to size in Table 4 we find that for micro start-ups there is a lower probability of survival, and that medium and large start-ups drive the estimates upwards. Also, it is possible that investing more affected survival chances and that the rate of relative investment for start-ups is biasing the estimates (later after we introduce the investment variable the size of effect for start-ups is considerably lower and the sign changes). The third variable, a dummy indicating at least 50% foreign ownership, implies that foreign companies had a higher probability of failure (15.7%). We assume it was easier for foreign investors to pull out their capital once things started going bad.

Observing by legal form agricultural firms (farms) and freelance occupations had higher probabilities of survival during the crisis, which isn’t surprising—they tend to be much less sensitive to crisis shocks. Firm size and ownership type estimates confirm the earlier non-parametric estimates, graphically summarised in Figs 4 and 5. We have added another new variable into the mix, representing the geographic county in which each firm was based. As expected in all of them firms were having a higher probability of failure during the crisis, implying that the shock was aggregate and hit the country as a whole, and was not region-specific. The only exceptions were the Splitsko-Dalmatinska County (no. 17) and the Dubrovacka County (no. 19), where firms had a higher probability of surviving the crisis. We attribute these exceptions to the fact that these two southern counties depend mostly on tourism, the demand for which was not that much affected during the crisis (at least relatively to other industries). Finally, we should note here that we also tested for industry dummies, specifically by using the industry classification in the transition matrix in (S1 and S2 Tables), but found no significant effect of the crisis on any industry. This further verifies our point that the crisis in Croatia was an aggregate shock, not an idiosyncratic one.

#### Investing vs non-investing firms

To test our hypothesis whether investing firms are better of than non-investing firms during recessionary periods we used the following empirical strategy. Since the first signal of the crisis was already noticeable in early 2008 and the first drop of Croatian GDP happened in the first two quarters of 2009, firms had one year at most, six months at least, to adapt their behaviour to the upcoming crisis i.e. to the anticipated shock. As observed in the data and regardless of the negative anticipated shock on the economy, some firms invested and some did not (note that our sample of investing vs non-investing has decreased to around 88,000 firms. This is because in 2007 and 2008 only 88,000 firms existed. Our full population of 144,000 firms are all firms that existed at some point from 2007 to 2015. Some failed, others were founded in the later years). We created a dummy variable that shows whether there were any investments in long-term assets in 2008. The reasons why we focused solely on long-term assets are multiple. Firstly, investment in long-term assets conditional on observing the negative shock indicates that the entrepreneur’s expected profit of investing is higher than expected profit of non-investing. Secondly, investment into long-term assets needs existing financial or credit power (access to finance), and a proper business strategy for investment projects. Thirdly, it represents a long-term investment meaning that it will have to endure the upcoming recessionary shock.

Obviously, the decision of whether to invest or not during 2008 was endogenous to firms and will be correlated with the probability of survival or specific firm level indicators. To remedy this potential endogeneity problem we used a matching method to match investing and non-investing firms into random samples based on several firm characteristics. The firm characteristics we use are size of assets, total revenues, total capital, and outstanding loans (Table 5). We restrained our analysis on only these characteristics to avoid the curse of dimensionality by using too many variables and layers. Basically, we divide the firms before the anticipated shock into two groups: investing and non-investing firms so that their mean values are almost the same. Furthermore, the whole population was divided into several layers to find firms that had similar predetermined characteristics (assets, sales, employees) but differed only by their investment strategy.

**Table 5:** Testing of post-matching characteristics between investing and non-investing firms.

	Micro Investing	Non-investing	Small Investing	Non-investing	Medium Investing	& Large Non-investing
Long-term assets	1.99	1.16	19.3	20.5	106	112
Short-term assets	1.87	1.18	18.7	16.5	79.2	63.2
Capital	0.625	0.561	6.09	6.71	6.84	5.35
Revenues	3.48	1.31	37.4	31.3	150	146
Loans	0.062	0.058	0.601	0.301	3.54	0.98
Frequency	25,650	59,286	2,676	1,141	786	129

Mean values are reported, the numbers are all in millions of kunas.

In Table 5 we provide a formal test of success of our matching procedure. The mean values across long-term assets, short-term assets, capital, revenues, and loans are almost the same compared across different size (at least 4 out of 5 in each case). We therefore reject the hypothesis of differences of means. Henceforth we can test the causal impact of investing during recession, since we have a random matching setup across investing and non-investing firms before the crisis.

**Fig 6:**
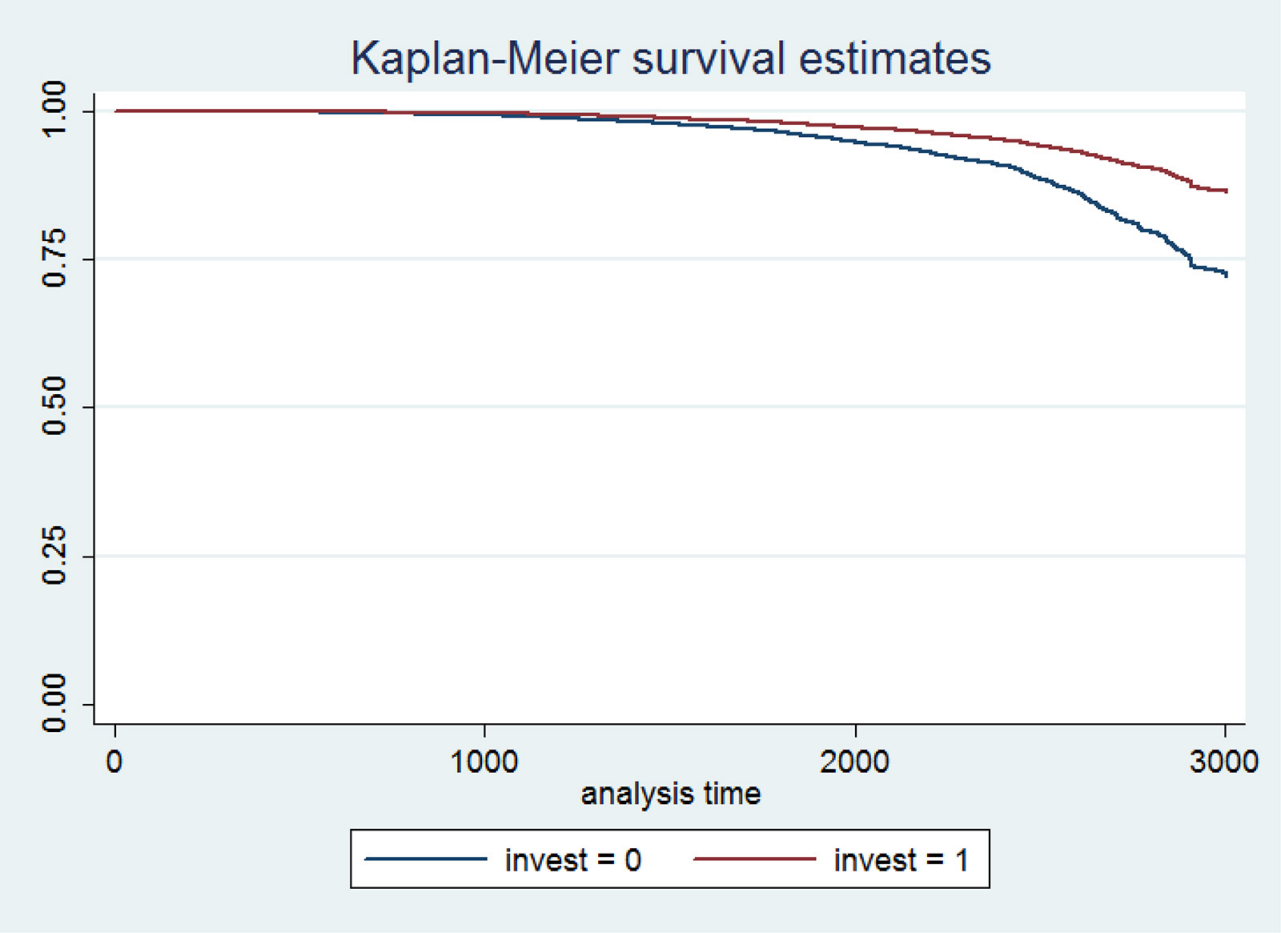
Kaplan-Meier survival estimate for investing and non-investing firms (micro firms).

We next focus on the survival analysis across different layers according to size. We start with micro firms and check if there was a difference in survival rates conditional on being an investing or a non-investing firm. The Kaplan-Meier survival estimates (Fig 6) show that there was a significant difference between investing and non-investing firms within this category. For micro firms it was clearly better to undertake long-term investments as a response to an anticipated shock. As shown in Table 6 (first column), investing micro firms had a 73.2% greater probability of survival.

**Table 6:** Cox proportional hazard model—survival rates based on firm size after matching.

Variable	Micro	Small	Medium and Large
Invest	-0.732 (0.018)***	-0.627 (0.116)***	0.012 (0.364)
Socialist origin	-0.121 (0.025)***	-0.439 (0.181)**	-0.194 (0.377)
Start-up	0.14 (0.017)***	0.065 (0.232)	-1.064 (0.744)
Foreign	0.199 (0.023)***	0.289 (0.169)	-0.398 (0.363)
**Legal form**			
Farms	-1.354 (0.5)***		
Freelance	-1.119 (0.119)***		
Craftsmen	0.44 (0.029)***	1.254 (0.191)***	2.788 (0.838)***
**Ownership type**			
State company in transformation	1.054 (0.286)***	2.57 (0.919)***	3.212 (0.88)***
State company where transformation has not started	1.12 (0.274)***	3.1 (0.772)***	1.03 (1.13)
Private since founding	0.446 (0.14)***	1.389 (0.584)**	0.807 (0.535)
Private after transformation	0.364 (0.166)**	0.886 (0.634)	-0.043 (0.631)
Cooperative ownership	0.418 (0.161)***	1.667 (0.918)	-38.78 (6.15e+08)
Mixed ownership with over 50% private capital	0.569 (0.198)***	1.52 (0.678)**	0.959 (0.591)
Mixed ownership with over 50% state capital	0.842 (0.205)***	1.212 (0.915)	0.559 (0.772)
Counties	YES	YES	YES
Observations	84,936	3817	1145
LR test (prob>Chi2)	3004.33 (0)	133.85 (0)	52.73 (0.012)

The table reports coefficients, not the hazard rates. Standard errors reported in parenthesis. *** denotes significance at 1%, ** at 5%, and * at 10%.

**Fig 7:**
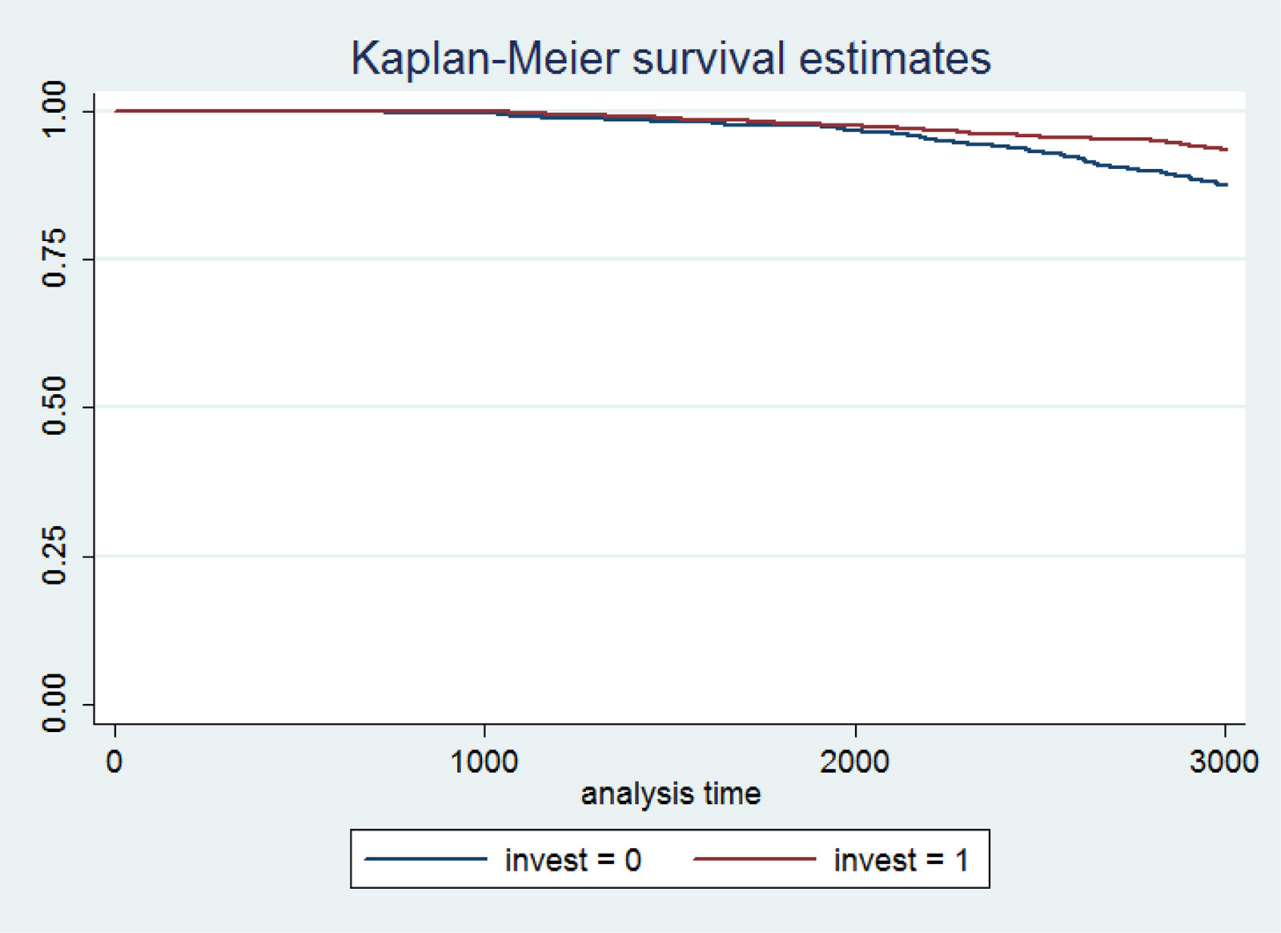
Kaplan-Meier survival estimate for investing and non-investing firms (small firms).

Next we analyze small firms were we found similarly encouraging results. The Kaplan Meier survival estimates (Fig 7) again show that firms which were investing in long-term assets had higher survival rates (62.7%) than those that chose not to invest.

**Fig 8:**
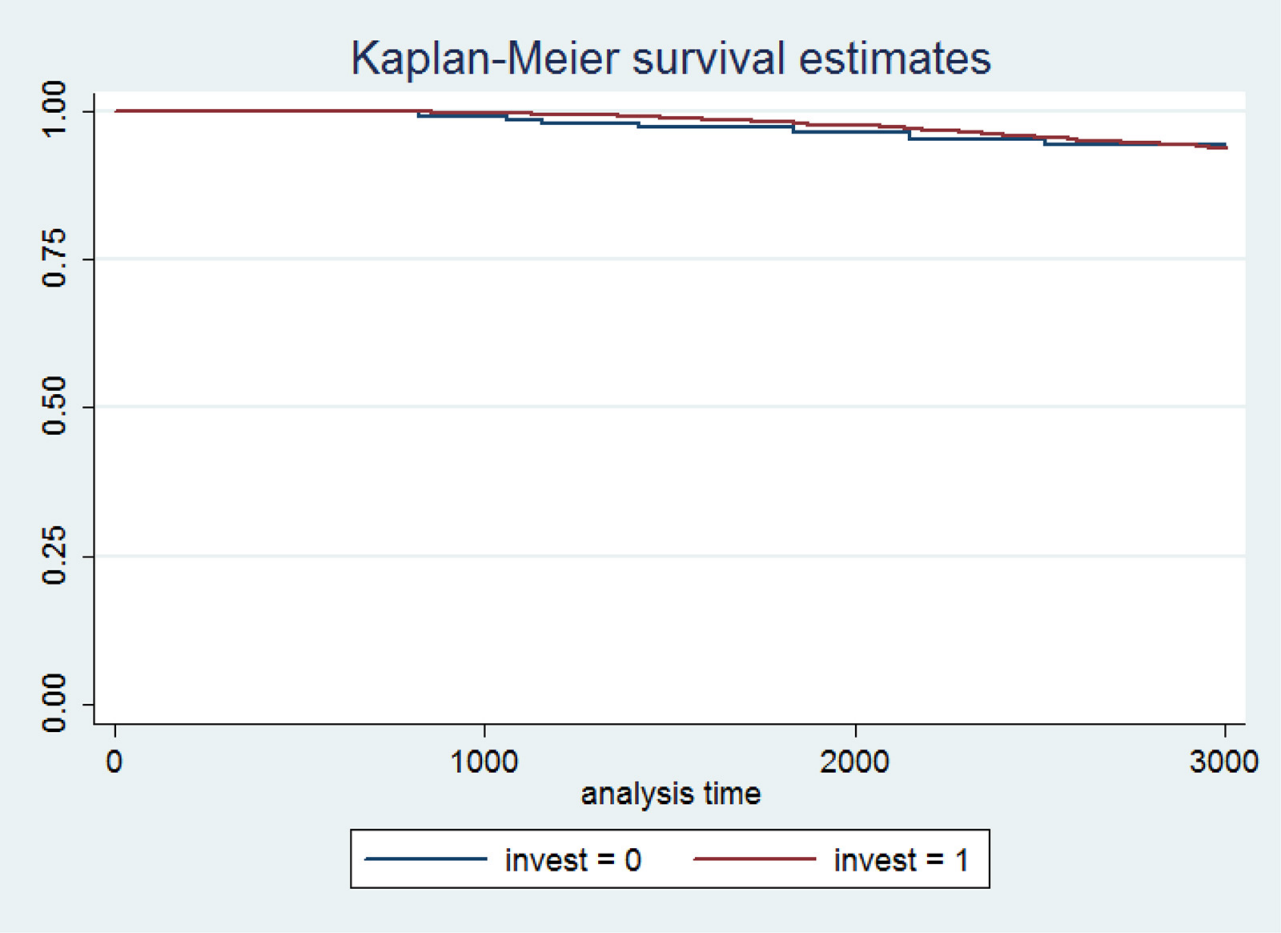
Kaplan-Meier survival estimate for investing and non-investing firms (medium and large firms).

The last part of this analysis focuses on medium and large firms where neither the Kaplan-Meier estimates (Fig 8) nor the Cox model support the earlier findings. Investment strategy does not seem to affect firm survival in case of medium and large firms. For micro and small firms investments are important as they imply increasing their business capacity and servicing a larger portion of the market. Large and medium firms have already reached their desired market share and size, meaning that their investment decisions will carry a lesser importance on their probability of survival than is the case with small and micro firms. In other words, investments relative to total assets will necessarily be lower for larger firms than for smaller ones, which diminishes their importance on survival of larger firms. Finally, the lack of significance could also be due to the lack of data, particularly when we separate the investing from non-investing firms. We would need a greater pool of medium and large firms to properly test this hypothesis. However, it is not possible to extend our database any further as it already contains the entire population of Croatian companies.

## Conclusion

The current literature states that economic agents should react to anticipated shocks by re-optimizing their current behaviour. We tested firms’ investment strategies conditional on observing a signal of an upcoming recession and found that the results support the hypothesis that survival is dependent on long-term investment. We compared similar firms according to size of assets, revenues, capital, and exposure to loans, where the only difference was a decision to invest as an anticipation of an upcoming economic shock. We found that for micro and small firms a dominant strategy was to invest. This increased their survival chances between 60 and 70% compared to firms that chose not to invest. For medium and large firms we find no effect between investment decisions and survival, probably because investments carried out by larger firms are relatively lower to their share of assets, making them a factor of lesser importance on determining survival. We also find that when there is a recession shock, firms change their investment strategy in those types of assets that have lower depreciation rates. Such behaviour is rational since this way they minimise their risk of investment by investing into long term and durable assets.

A possible normative implication of our findings is to advise small and micro firms not to delay long-term investments if they anticipate a crisis coming. Engaging in such investments prior to the expected shock sends a signal of confidence that firms are able to withstand the upcoming shock. This could create a positive feedback effect in which if less firms fail, they lay off less workers during recession periods, which means the crisis shock could be significantly less damaging for the economy’s aggregate demand, and that the recovery could occur faster. From a policy perspective governments can use this to help small and micro firms gain access to capital to initiate investments before the crisis hits.

## Supporting Information

S1 Table Between-industry transition probability matrix (part 1). (PDF)

S2 Table Between-industry transition probability matrix (part 2). (PDF)

S1 Dataset S1_Dataset.dta. (DTA)
