# Supplementary material for: To Invest or Not to Invest, That Is the Question: Analysis of Firm Behavior under Anticipated Shocks
Source: PLoS One. 2016 Aug 10;11(8):e0158782. doi: 10.1371/journal.pone.0158782 (PMC4979903; doi:10.1371/journal.pone.0158782)
Supplement: S2 Table — (PDF) [file pone.0158782.s002.pdf]

Supporting Information

To Invest or Not to Invest, That is the Question: Analysis of Firm Behavior Under Anticipated Shocks

Dejan Kovac[1-4], Vuk Vukovic[2-4*], Nikola Kleut[4-5], Boris Podobnik[2-4,6-7]

1 CERGE-EI, A joint workplace of the Center for Economic Research and Graduate Education, Charles University, Prague, and the Economics Institute of the Academy of Sciences of the Czech Republic, Prague, Czech Republic
2 Luxembourg School of Business, Luxembourg, Grand-Duchy of Luxembourg
3 Department of Economics, Zagreb School of Economics and Management, Zagreb, Croatia.
4 Adriatic Economic Association, Zagreb, Croatia
5 Zenlab d.o.o., Zagreb, Croatia
6 Faculty of Economics, University of Ljubljana, Ljubljana, Slovenia.
7 Faculty of Civil Engineering, University of Rijeka, Rijeka, Croatia.

* Corresponding author: vuk.vukovic@zsem.hr

Appendix

Table S2. Between-industry transition probability matrix (part 2)

	Finance	Real estate	Science	Administration	Public service	Education	Health	Arts	Other
Finance	85.12	1.09	5.47	1.53	0.00	0.00	0.22	0.00	0.00
Real estate	0.00	92.49	0.68	0.54	0.00	0.00	0.07	0.07	0.07
Science	0.01	0.27	95.50	0.37	0.02	0.09	0.03	0.05	0.05
Administration	0.09	0.75	1.36	91.30	0.04	0.13	0.00	0.18	0.26
Public Service	0.00	0.00	41.67	2.08	54.17	0.00	2.08	0.00	0.00
Education	0.00	0.00	1.08	0.92	0.00	95.84	0.00	0.15	0.00
Health	0.00	0.43	0.43	0.14	0.00	1.01	96.53	0.00	0.29
Arts	0.00	0.00	1.27	0.18	0.00	0.00	0.54	91.67	0.18
Other	0.00	0.36	1.51	0.07	0.00	0.14	0.07	0.29	92.33
